# The Anatomic–Atraumatic–Anterior Reconstructive Technique (A-A-ART) for total hip arthroplasty: A step-by-step pictorial guide to a minimally invasive approach for hip osteoarthritis

**DOI:** 10.1051/sicotj/2026071

**Published:** 2026-10-26

**Authors:** Angelo V. Vasiliadis, Vasileios Giovanoulis, Rigas Santas, Theofilos Dimitriadis, Cécile Batailler, Sébastien Lustig

**Affiliations:** 1 Univ Lyon, Claude Bernard Lyon 1 University, IFSTTAR, LBMC UMR_T9406 Lyon France; 2 Department of Orthopaedics Surgery and Sports Medicine, FIFA Medical Center of Excellence, Croix-Rousse Hospital, Hospices Civils de Lyon, Lyon North University Hospital Lyon France; 3 Department of Orthopaedic Surgery, St. Luke’s Hospital 55236 Panorama-Thessaloniki Greece; 4 Department of Orthopaedic Surgery, Bioclinic 54622 Thessaloniki Greece

**Keywords:** Total hip arthroplasty, Anterior approach, Surgical technique

## Abstract

Total hip arthroplasty remains one of the most successful orthopedic procedures for the treatment of hip osteoarthritis. Among contemporary surgical approaches, the direct anterior approach has gained increasing popularity due to its intermuscular and internervous plane, which aims to minimize soft-tissue trauma and potentially reduce instability. This article provides a step-by-step pictorial overview of the Anatomic–Atraumatic–Anterior Reconstructive Technique (A-A-ART) performed without a traction table and combined with dual-mobility articulation. Key technical steps are described, including patient positioning, capsular management, acetabular preparation using anatomical landmarks, controlled femoral exposure, and implant positioning. Particular emphasis is placed on progressive capsular release, protection of the anterior acetabular horn, and prevention of varus stem alignment. When performed in a standardized and reproducible manner, the A-A-ART technique allows reliable limb length control, stable component orientation, and preservation of periarticular structures, offering a safe and effective strategy for the surgical management of hip osteoarthritis.

## Introduction

Total hip arthroplasty (THA) is considered one of the most successful surgical procedures of the 20th century, owing to its excellent clinical outcomes, including pain reduction, restoration of mobility and high satisfaction in patients with hip joint disorders [1]. Notably, more than 1 million THA procedures are performed annually worldwide, and this number is expected to increase further with the aging of the global population [2].

In this context, multiple surgical approaches for THA have been developed in an effort to optimize outcomes and reduce complications. The posterior, lateral, and anterolateral approaches are the most commonly used techniques, with several variations proposed by different authors, each offering specific advantages and disadvantages in terms of surgical exposure, joint stability, muscle preservation, and post-operative function [3–5]. More recently, minimally invasive and muscle-sparing techniques, such as the direct anterior approach (DAA), often utilizing a traction table and intra-operative fluoroscopy, have gained increasing popularity, with the aim of minimizing soft-tissue trauma and facilitating faster functional recovery [6, 7]. This approach is considered to be associated with a lower risk of dislocation due to preservation of the posterior capsule and surrounding musculature, while respecting native anatomy and minimizing tissue damage.

The present step-by-step pictorial overview aims to familiarize young surgeons with a standardized technique of THA performed through the DAA and to provide a practical guide to its key technical steps. Given its intermuscular and internervous pathway, its emphasis on capsular preservation, and its reconstructive focus on restoring hip biomechanics while minimizing soft-tissue trauma, this technique may be conceptually framed as an Anatomic–Atraumatic–Anterior Reconstructive Technique (A-A-ART). This terminology does not denote a new surgical approach, but rather highlights a structured, tissue-respecting anatomic philosophy within the established anterior approach to the hip.

## Surgical technique

### Patient positioning

The procedure is most commonly performed under spinal anesthesia, which provides optimal muscle relaxation. The patient is positioned supine on a standard operating table, which remains unmodified and stationary throughout the procedure. Both lower limbs are draped separately to allow free manipulation during femoral preparation, and limb lengths are compared at the start of the operation. Pre-operative radiographs are used for planning, with anticipated limb length adjustment of the operative side determined in advance. The upper limb ipsilateral to the operative hip is placed across the chest and secured.

### Surgical approach

The skin incision is made at the middle of the line connecting the anterior superior iliac spine and the tip of the greater trochanter (Figure 1A). It is centered on the tensor fasciae latae muscle, with its length adjusted according to the patient’s anatomy. The subcutaneous tissue is incised with electrocautery to expose the tensor fasciae latae (Figure 1B). The aponeurosis of the tensor fasciae latae is then incised longitudinally, in line with muscle fibers, using a scalpel (Figure 1C). The aponeurosis is carefully detached from the underlying muscle by blunt digital (index finger) dissection, and perforating veins are coagulated as required. A counter-angled retractor is placed superior to the femoral neck to maintain the interval between the tensor fasciae latae and the sartorius muscle. The tensor fasciae latae is retracted laterally using an additional retractor (Figure 1D).

**Figure 1:**
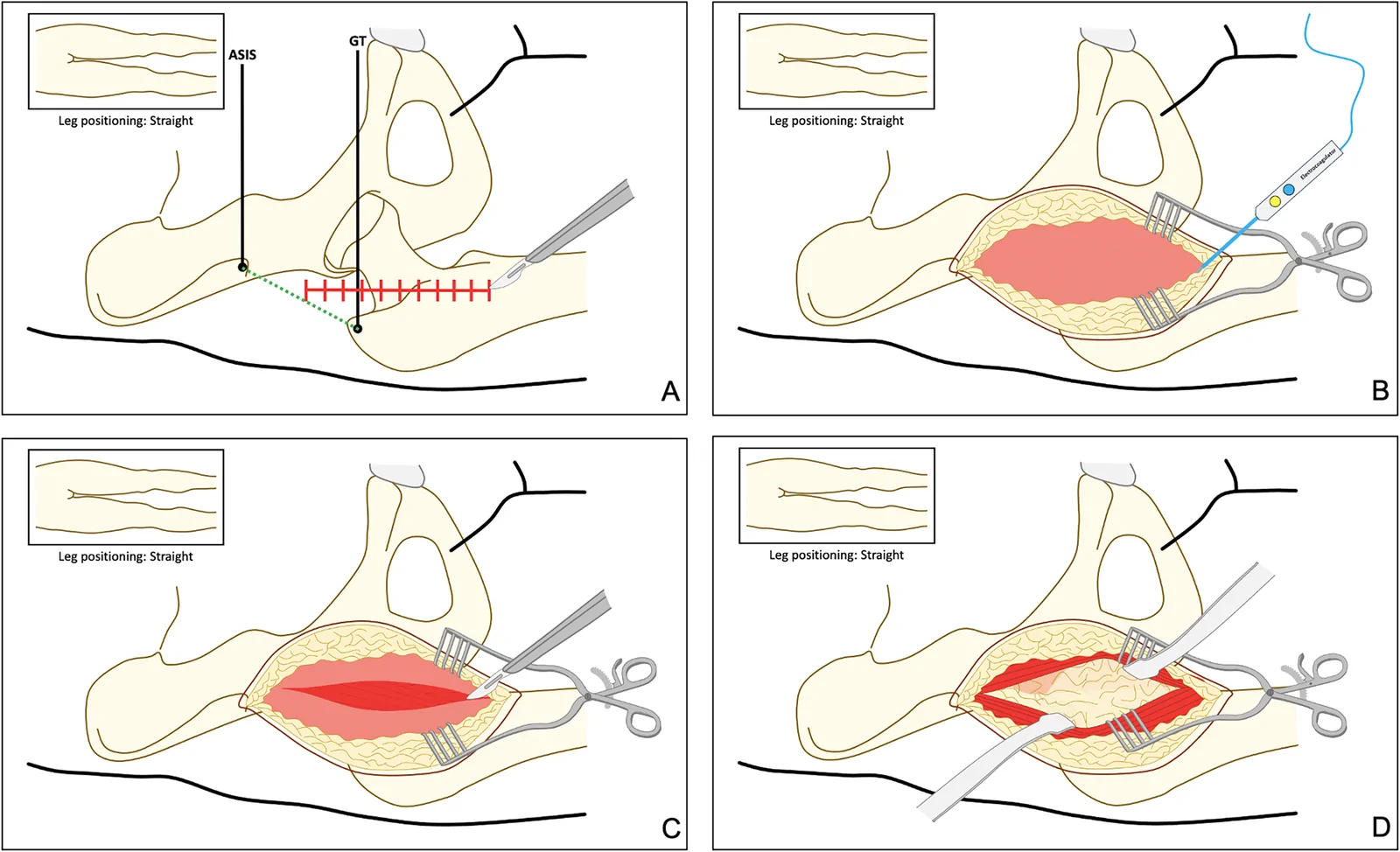
Illustrations show the skin incision, which is made at the middle of the line connecting the anterior superior iliac spine and the tip of the greater trochanter (abbreviations: ASIS, anterior superior iliac spine; GT, greater trochanter) (A), the subcutaneous tissue, which is incised with electrocautery to expose the tensor fasciae latae (B), the aponeurosis of the tensor fasciae latae, which is incised longitudinally, in line with muscle fibers, using a scalpel (C) and the placement of a counter-angled retractor superior to the femoral neck to maintain the interval between the tensor fasciae latae and the sartorius muscle, with the tensor fasciae latae retracted laterally using an additional retractor (D).

The anterior circumflex pedicle lies beneath the innominate fascia. This fascia is carefully incised to allow placement of a counter-angled retractor along the inferior aspect of the femoral neck and to expose the anterior circumflex pedicle, which is then meticulously cauterized (Figure 2A). The rectus femoris muscle is dissected off the capsule using a perforated periosteal elevator. This dissection permits placement of a counter-angled retractor against the anterior horn of the acetabulum, thereby fully exposing the anterior surface of the capsule through medial retraction of the sartorius and rectus femoris muscles (Figure 2B). Strict control of the anterior acetabular horn is essential to prevent any anterior displacement of the acetabular cup. The capsule is then incised in a reverse U-shape fashion, beginning with the inferior flap (Figure 2C), and the retractors are progressively repositioned intra-capsularly (Figure 2D). At this stage, a capsulectomy is performed in order to facilitate femoral exposure. The superior capsule should be released as extensively as possible along the medial aspect of the greater trochanter to optimize femoral exposure during femoral preparation (Figure 2E). A gauze pad may be temporarily placed intra-capsularly posterior to the femoral neck to protect the posterior capsule, the pelvitrochanteric muscles and the posterior circumflex pedicle.

**Figure 2:**
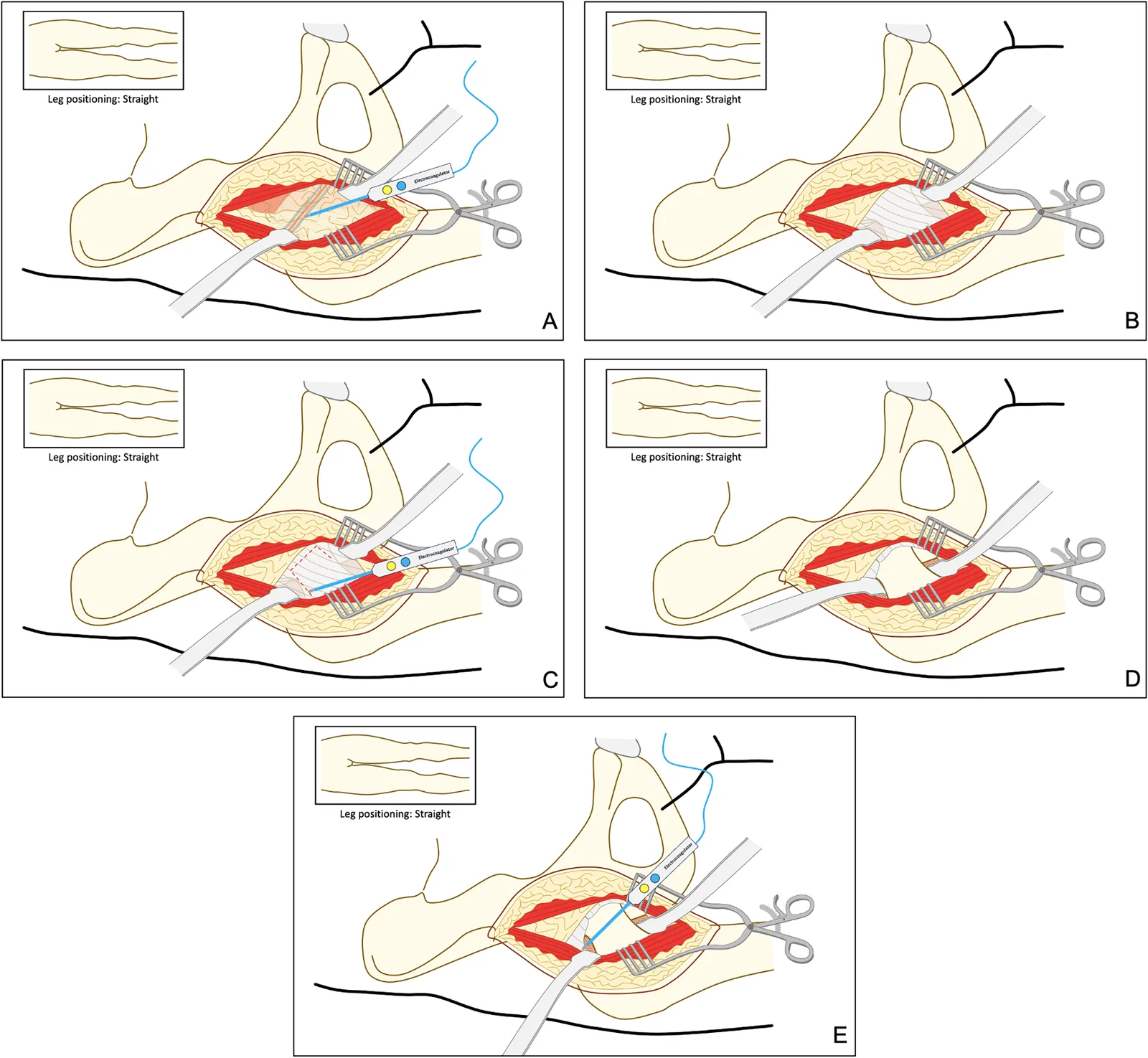
Illustrations show careful placement of retractors along the inferior and lateral aspects of the femoral neck, facilitating exposure and meticulous cauterization of the anterior circumflex pedicle (A), final placement of a counter-angled retractor against the anterior horn of the acetabulum, fully exposing the anterior capsule through medial retraction of the sartorius and rectus femoris muscles (B), incision of the capsule in a reverse U-shape fashion, beginning with the inferior flap (C), progressive intra-capsular repositioning of the retractors (D) and extensive release of the superior capsule along the medial aspect of the greater trochanter to optimize femoral exposure during preparation (E).

### Femoral neck osteotomy

The osteotomy is performed without hip dislocation, maintaining, if possible, in slight internal rotation to allow controlled “unrolling” of the femoral neck. A femoral rasp is positioned vertically above the femur and the blade of the oscillating saw is aligned against the rasp to determine the cutting direction (Figure 3A). This orientation, together with the apex of the greater trochanter, which is palpated beforehand, defines the plane of the femoral neck osteotomy. The osteotomy level is adjusted according to the preoperative planning. The femoral neck is sectioned in two stages to facilitate safe extraction of the femoral head and to minimize the risk of injury to the tensor fasciae latae (Figure 3B). First, a bone slice is removed, followed by extraction of the femoral head using a corkscrew (Figure 3C).

**Figure 3:**
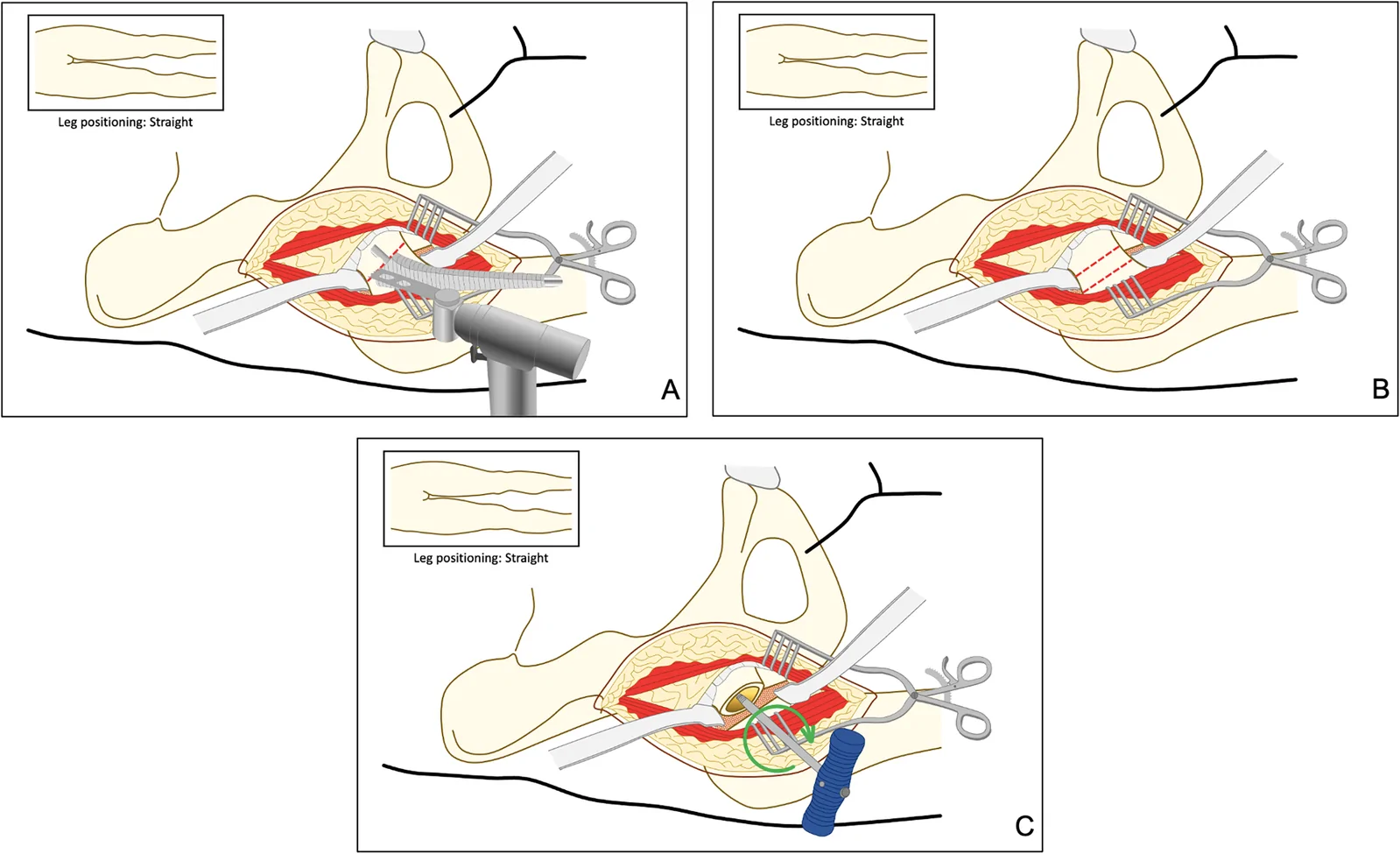
Illustrations show a femoral rasp positioned vertically above the femur, with the blade of the oscillating saw aligned against the rasp to guide the cutting direction (A), the two marked stages on the femoral neck, facilitating safe extraction of the femoral head while minimizing the risk of injury to the tensor fasciae latae (B), and the femoral head being extracted using a corkscrew (C).

### Acetabular exposure

The retractors are then repositioned to expose the acetabulum. Medially, a retractor is placed against the anterior surface of the acetabulum. Excessive traction on this retractor must be avoided, as it may result in injury to the rectus femoris muscle or weakening of the anterior acetabular horn. A second retractor is positioned beneath the transverse acetabular ligament. Finally, a third retractor is placed posterior to the acetabular horn, retracting the femur out of the operative field while protecting the tensor fasciae latae (Figure 4A). This retractor configuration typically provides excellent visualization of the acetabulum and allows precise control of the bony contours.

**Figure 4:**
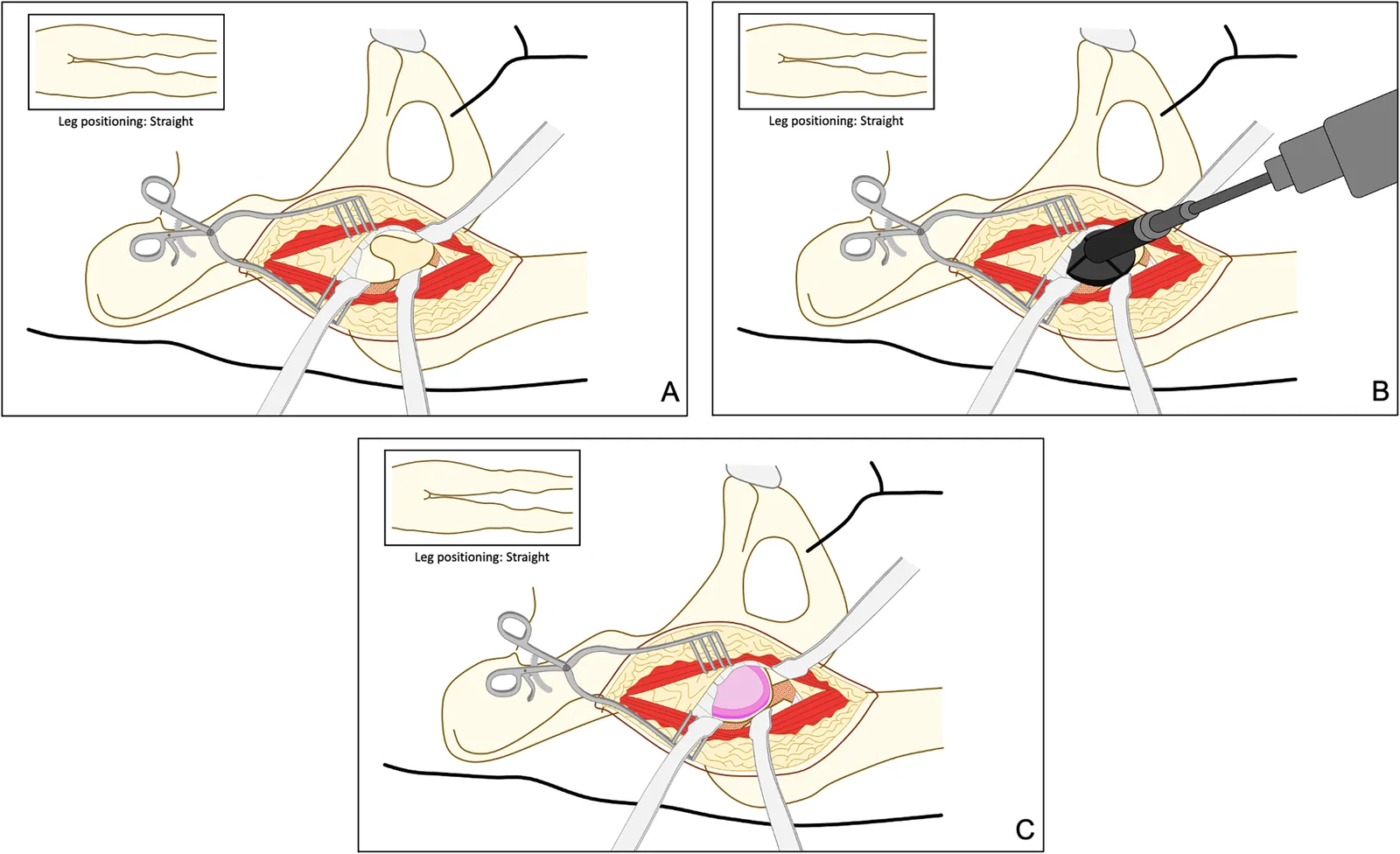
Illustrations show the final retractor configuration, which typically provides excellent visualization of the acetabulum and allows precise control of the bony contours (A), acetabular reaming performed using a standard straight reamer of progressively increasing diameters until the target size for the definitive implant is achieved, while maintaining consistent alignment along the anteversion axis (B) and implantation of the definitive cup using a straight impactor following thorough irrigation of the acetabulum (C).

The acetabulum is prepared by excising the labrum, the ligamentum teres, and any residual anterior capsule. Reaming is performed using a standard straight reamer (Figure 4B). If the posterior capsule appears tight, it may be gently thinned; this release also facilitates subsequent femoral exposure. The initial reamer diameter is selected to be 6 mm smaller than the planned final implant diameter. Reamer orientation is guided by the transverse acetabular ligament and the anterior acetabular horn, which serve as anatomical landmarks to achieve approximately 40°–45° of abduction and appropriate anteversion adapted to the patient’s acetabular anatomy. Reaming is then performed with progressively increasing diameters until the target size for the definitive implant is reached, while maintaining consistent alignment along the anteversion axis.

A trial cup is positioned within the acetabulum. Anteversion is assessed using the transverse acetabular ligament and the acetabular rim as reference landmarks. Care must be taken to ensure that the acetabular component does not extend anterior to the anterior horn, in order to avoid potential iliopsoas impingement. Appropriate implant sizing is confirmed by the stable seating of the trial component, which is then removed. After thorough irrigation of the acetabulum, the definitive cup is implanted using a straight impactor (Figure 4C). Secure retention of the impactor is essential, as insertion of the implant into the bony acetabulum places significant stress on the acetabular carrier, which may disengage if retention is inadequate. The target orientation is approximately 40° of abduction, with anteversion adapted to the patient’s anatomy.

### Femoral preparation

Exposure of the femur is the most demanding part of this procedure when an extension table is unavailable. The initial release is made in the medial capsule, aiming for a complete release of the tight pubofemoral ligament down to the lesser trochanter while avoiding damage to the iliopsoas tendon. It is necessary to allow a few extra minutes to properly expose the femur. A counter-angled retractor is placed anterior to the femur, medially retracting the muscles and protecting the acetabulum. A femoral elevator is positioned behind the greater trochanter, thus slightly elevating the femur. A Lambotte hook is placed in the medullary canal, allowing for progressive traction on the femur. This traction moves the greater trochanter anterior to the acetabulum while simultaneously putting tension on the antero-superior and postero-medial capsules. The capsule is then progressively released in a clockwise manner, at the level of the medial aspect of the greater trochanter using electrocautery to facilitate elevation and external rotation of the femur (Figure 5A). This maneuver results in a gradual elevation of the femur, controlled by the femoral elevator.

**Figure 5:**
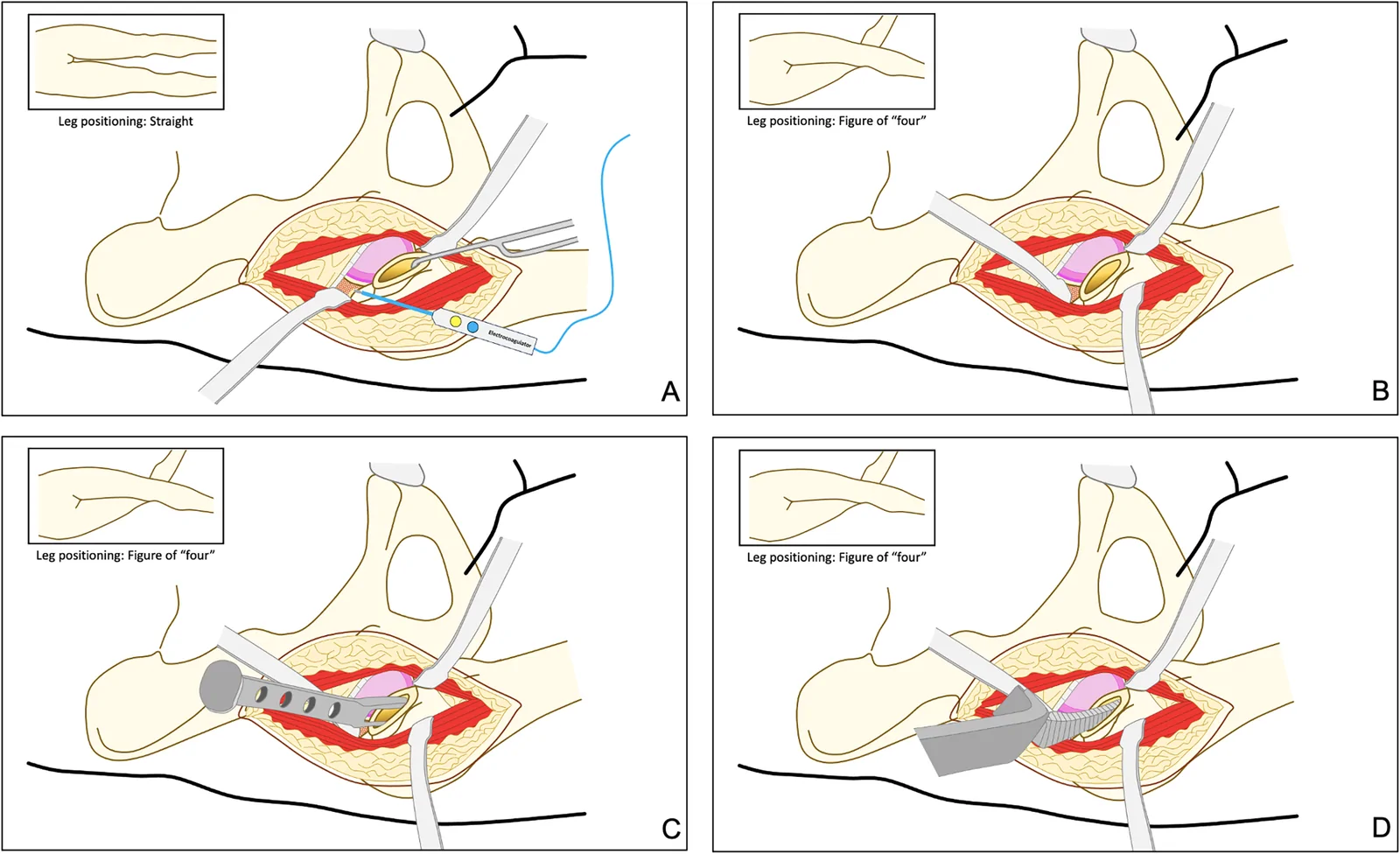
Illustrations show anterior retractor placement with femoral elevation and traction using a femoral elevator and Lambotte hook, moving the greater trochanter anterior to the acetabulum and tensioning the anterosuperior capsule for progressive release along the medial aspect of the greater trochanter using electrocautery (A), exposure of the femoral canal by positioning the operated lower limb beneath the contralateral limb, producing approximately 20°–30° of femoral adduction and external rotation (B), preparation of the femoral canal with rasp insertion using a proximal starter to minimize the risk of varus stem positioning (C) and a diaphyseal rasp used to gently prepare the femoral canal for final femoral implantation (D).

The femoral canal is exposed by placing the operated lower limb beneath the contralateral limb, producing approximately 20°–30° of femoral adduction and external rotation (Figure 5B). This maneuver should not be performed until the greater trochanter has moved anterior to the acetabulum, as premature manipulation may result in fracture of the greater trochanter. Exposure of the femoral shaft is then completed by progressively releasing the capsule as required, up to the pelvis–trochanteric muscles, which are preserved during this procedure. Clear visualization of the femoral neck is essential before proceeding further with the operation.

The femoral canal is prepared for rasp insertion using a proximal starter to minimize the risk of varus stem positioning (Figure 5C). A diaphyseal rasp with a “rat’s tail” angulation is then gently introduced to prepare the medial aspect of the greater trochanter, followed by the sequential insertion of rasps of increasing size (Figure 5D). The use of a dedicated double-offset rasp holder helps prevent interference with retractors and the anterior superior iliac spine while maintaining proper alignment with the femur. Once the trial rasp corresponding to the preoperative plan is in place, a cortical burr is used to equalize the level between the bone and the implant. A trial head is then inserted, and the hip is reduced with the dual-mobility insert already in place. Leg length and hip stability are assessed through extreme ranges of motion. Fluoroscopy may be used to confirm adherence to the preoperative plan, and adjustments to neck length or stem insertion depth can be made if necessary.

### Stem implantation and final reduction

The final stem is then impacted (Figure 6A), and the depth of insertion is verified before positioning and impacting the final component (head and polyethylene). Using a reducer, a plastic shoehorn (Figure 6B), type instrument that displaces the rectus femoris muscle and guides the component into the acetabular cup the joint is reduced (Figure 6C), while longitudinal traction is applied along the limb axis by the surgical assistant. This maneuver can be delicate and requires optimal muscle relaxation. Final limb length assessment and range-of-motion testing are subsequently performed.

**Figure 6:**
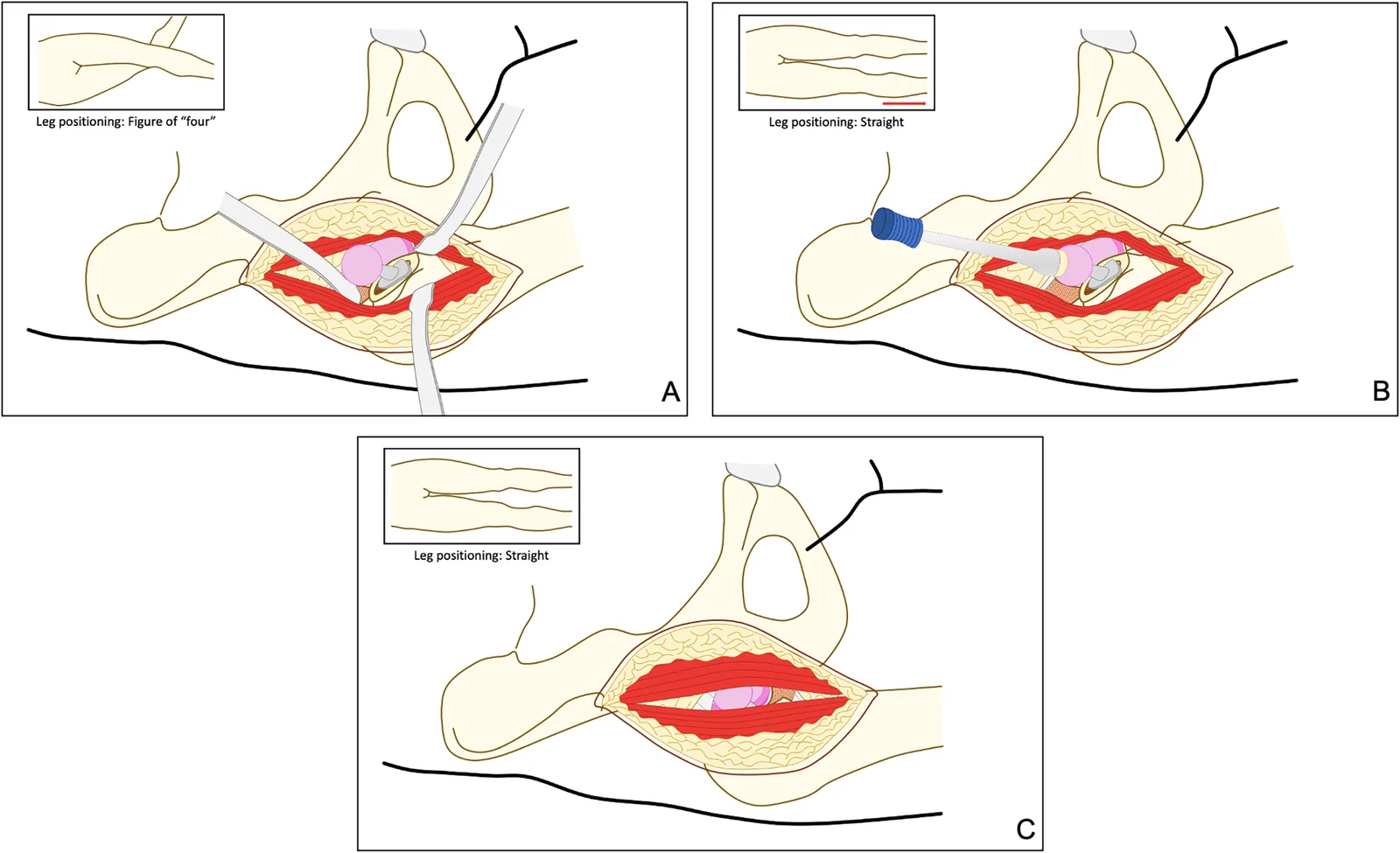
Illustrations show the final stem impaction (A), the effort of joint reduction using a reducer (plastic shoehorn) (B), and the femoral component positioned within the acetabular cup after reduction of the hip joint (C).

### Wound closure

If the capsule has been preserved, it is closed with simple sutures. The fascia of the tensor fasciae latae is then closed using an absorbable suture in a continuous pattern to restore soft tissue integrity and ensure a watertight closure (Figure 7). The subcutaneous tissues and skin are subsequently closed in the usual anatomical manner.

**Figure 7:**
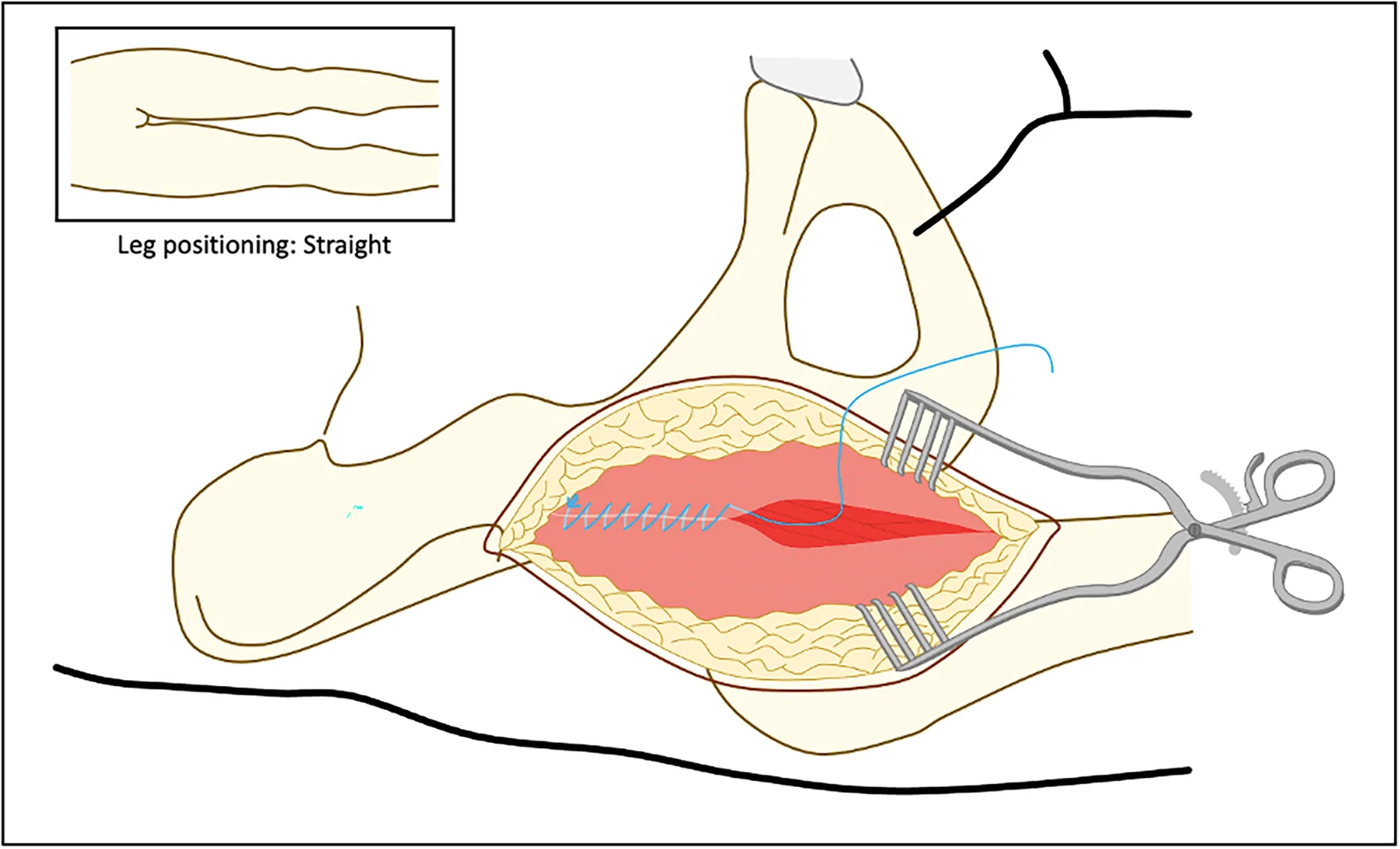
Illustration shows closure of the fascia of the tensor fasciae latae using a continuous pattern with absorbable suture.

## Discussion

This article describes a standardized, step-by-step direct anterior technique for THA, termed A-A-ART, performed without a traction table and combined with dual-mobility articulation. The DAA has gained increasing popularity due to its intermuscular and internervous interval, thereby minimizing muscle injury and preserving peri-articular soft tissues [6, 7]. In contrast to traction table-assisted techniques, A-A-ART relies on controlled manual limb positioning and progressive soft-tissue release, avoiding excessive traction forces while facilitating safe femoral and acetabular exposure. This anatomical and tissue-sparing philosophy aims to preserve native biomechanics, reduce iatrogenic soft-tissue trauma, and potentially lower the risk of post-operative instability. Several studies have reported comparable or lower dislocation rates with the anterior approach than with posterior approaches, although functional advantages appear to be most evident during the early post-operative period [7, 8]. The present A-A-ART technique further emphasizes meticulous respect for anatomical planes, graduated capsular release, controlled limb manipulation and precise implant positioning to optimize stability, restore hip biomechanics and enhance procedural reproducibility.

Although this technique requires specific retractors and offset rasp holders, it eliminates the need for a traction table and allows direct intra-operative control of limb length and hip stability. However, surgeons transitioning from another approach must adapt to a different anatomical perspective and surgical workflow. A learning curve is unavoidable, with higher complication rates reported during the early phase of adoption, particularly with regard to femoral exposure and intra-operative fractures [8, 9]. For this reason, careful patient selection during the initial experience is recommended, avoiding very muscular individuals, marked coxa vara with short femoral necks, and severe osteoporosis. Progressive capsular release, strict respect of the anterior horn during acetabular preparation, and prevention of varus stem positioning represent critical technical principles. The key surgical steps and practical recommendations for surgeons adopting this technique are summarized in Table 1.

**Table 1:** Key steps and practical recommendations for surgeons learning the direct anterior approach.

Surgical step	Technical objective	Pitfalls	Practical tips
Patient positioning	– Stable supine positioning – Allow intra-operative limb manipulation	– Inadequate draping limiting femoral exposure – Poor limb assessment	– Drape both legs separately – Compare limb lengths – Ensure muscle relaxation
Skin incision and interval plane	– Correctly identify intermuscular plane (TFL and sartorius)	– Entering the wrong plane leading to bleeding and poor exposure	– Take time to clearly identify the TFL – Incise fascia longitudinally – Avoid aggressive dissection
Vascular control	– Identify and coagulate the ascending branch of LFCA	– Inadequate hemostasis causing hematoma and poor visualization	– Systematically search and cauterize the vascular branch
Capsulotomy	– Adequate exposure of femoral neck and acetabulum	– Inadequate capsular release leading to difficult femoral exposure	– Perform progressive capsular release – Free the superior capsular medially at the GT
Femoral neck osteotomy	– Accurate osteotomy according to pre-operative planning	– Incorrect osteotomy level	– Use a rasp as a guide for saw orientation – Perform osteotomy in two steps
Acetabular exposure	– Achieve stable and safe retractor positioning	– Excessive force on anterior horn leads to rectus femoris injury	– Avoid levering on the anterior rim – Position retractors under direct vision
Cup orientation	– Correct inclination (40–45°) – Anatomical anteversion (10–15°)	– Anterior overhang causing iliopsoas impingement	– Use transverse acetabular ligament as a reference – Confirm orientation with fluoroscopy
Femoral exposure	– Mobilize femur safely without trochanteric fracture	– Forcing exposure before adequate capsular release	– Ensure GT passes anterior to acetabulum before adduction/external rotation
Canal preparation	– Avoid varus stem positioning	– Inadequate canal entry – Incorrect rasp alignment	– Use a starter reamer – Consider minimal trochanteric cortical trimming – Use double-offset rasp holder
Trial reduction	– Assess stability, offset, and limb length	– Difficult reduction indicating improper soft tissue balance or implant selection	– Use shorter neck during trials – Reduce trial head alone in dual-mobility systems
Definitive reduction	– Achieve atraumatic final reduction	– Excessive force due to insufficient relaxation	– Ensure optimal anesthesia – Use reducer device
Wound closure	– Restore soft-tissue planes – Promote rapid recovery	– Inadequate fascia repair	– Repair TFL fascia carefully

Abbreviations: TFL, tensor fascia latae; LFCA, lateral femoral circumflex artery; GT, greater trochanter.

With increasing surgical experience, the anterior approach performed without a traction table can reliably accommodate modern implant designs, including dual-mobility cups, which have demonstrated a reduced risk of post-operative instability, particularly in elderly or high-risk patients [9]. Moreover, the supine positioning facilitates intra-operative limb length assessment and permits sequential bilateral procedures under the same anesthesia without repositioning, in accordance with enhanced recovery protocols. Given its muscle-sparing, intermuscular and internervous nature, combined with meticulous capsular preservation and a reconstructive focus on restoring native hip biomechanics, this technique may be conceptually framed under the term. This terminology does not introduce a new surgical approach, but rather reflects a structured, tissue-respecting philosophy applied within the established DAA. By emphasizing atraumatic soft-tissue handling and reproducible biomechanical reconstruction through an anterior corridor, the term A-A-ART underscores the principles that underpin the described method while remaining grounded in the anatomical foundations of the DAA.

## Conclusion

The anterior approach to THA performed without a traction table represents a reproducible and tissue-preserving, anatomic technique when executed with meticulous capsular management, precise implant orientation, and controlled femoral exposure. Its intermuscular and internervous pathway, combined with dual-mobility articulation, offers reliable stability and facilitates early functional recovery. Framing this method as the A-A-ART underscores its reconstructive intent and soft-tissue-respecting philosophy, while remaining grounded in the established principles of the DAA. With appropriate patient selection and awareness of the learning curve, this strategy provides a safe and effective option for the surgical treatment of hip osteoarthritis.

## Data Availability

Not applicable to this study.
